# Regulation of MicroRNAs, and the Correlations of MicroRNAs and Their Targeted Genes by Zinc Oxide Nanoparticles in Ovarian Granulosa Cells

**DOI:** 10.1371/journal.pone.0155865

**Published:** 2016-05-19

**Authors:** Yong Zhao, Lan Li, Ling-Jiang Min, Lian-Qin Zhu, Qing-Yuan Sun, Hong-Fu Zhang, Xin-Qi Liu, Wei-Dong Zhang, Wei Ge, Jun-Jie Wang, Jing-Cai Liu, Zhi-Hui Hao

**Affiliations:** 1 College of Chemistry and Pharmaceutical Sciences, Qingdao Agricultural University, Qingdao, 266109, P. R. China; 2 Key Laboratory of Animal Reproduction and Germplasm Enhancement in Universities of Shandong, Qingdao, 266109, P. R. China; 3 College of Animal Science and Technology, Qingdao Agricultural University, Qingdao, 266109, P. R. China; 4 State Key Laboratory of Reproductive Biology, Institute of Zoology, Chinese Academy of Sciences, Beijing, 100101, P. R. China; 5 State Key Laboratory of Animal Nutrition, Institute of Animal Sciences, Chinese Academy of Agricultural Sciences, Beijing, 100193, China; Institute for Materials Science, GERMANY

## Abstract

Zinc oxide (ZnO) nanoparticles (NPs) have been applied in numerous industrial products and personal care products like sunscreens and cosmetics. The released ZnO NPs from consumer and household products into the environment might pose potential health issues for animals and humans. In this study the expression of microRNAs and the correlations of microRNAs and their targeted genes in ZnO NPs treated chicken ovarian granulosa cells were investigated. ZnSO_4_ was used as the sole Zn^2+^ provider to differentiate the effects of NPs from Zn^2+^. It was found that ZnO-NP-5 μg/ml specifically regulated the expression of microRNAs involved in embryonic development although ZnO-NP-5 μg/ml and ZnSO_4_-10 μg/ml treatments produced the same intracellular Zn concentrations and resulted in similar cell growth inhibition. And ZnO-NP-5 μg/ml also specifically regulated the correlations of microRNAs and their targeted genes. This is the first investigation that intact NPs in ZnO-NP-5 μg/ml treatment specifically regulated the expression of microRNAs, and the correlations of microRNAs and their targeted genes compared to that by Zn^2+^. This expands our knowledge for biological effects of ZnO NPs and at the same time it raises the health concerns that ZnO NPs might adversely affect our biological systems, even the reproductive systems through regulation of specific signaling pathways.

## Introduction

Tons of Zinc Oxide (ZnO) nanoparticles (NPs) are produced annually in the world [1]. ZnO NPs are widely used in medical, industrial, and household applications [1–8], as anti-virus and anti-bacteria [9–11] agents due to their small size and excellent biological properties. Therefore the release of ZnO NPs from consumer and household products into the environment is much possible and they may pose potential threat to the animals and humans [12–16]. Many studies have reported that ZnO NPs caused adverse effects on human cells [17–21] through creating intracellular oxidative stress to induce apoptosis [22,23], and caused toxicity on mouse, rat, zebrafish embryos, *Daphnia magna* [11–16]. Furthermore, because of the extensive application of ZnO NPs, it is speculated that the human body may be intentionally or unintentionally exposed to ZnO NPs through several possible routes, including oral ingestion, inhalation, intravenous injection, and dermal penetration [24]. And uptake of ZnO NPs by the gastrointestinal tract is considered as one of the very important routes [24].

Small noncoding RNAs (~20–30 nucleotides (nt)): microRNAs (miRNAs), Piwi-interacting small RNAs (piRNAs) and endogenous small interfering RNAs (endo-siRNAs) are the three major classes of endogenous small RNAs identified in organisms [25, 26]. These small regulatory RNAs play very important roles in gene regulation in the vertebrate genome [27]. One group of these small RNAs, microRNAs are small noncoding molecules ranging in size from 19 to 24 nucleotides (nt). It has been estimated that microRNAs could regulate the expression of 20% to 30% of the genes in a genome. MicroRNAs play vital roles in stem cell reprogramming [28], diverse developmental processes especially embryonic development [29], bone formation [30] and ovarian functions [31]. MicroRNAs have regulatory functions not only in normal development, physiology [32–34] and evolution but also in disease [35, 36]. In addition to human, mouse or rat, microRNAs have also been investigated in farm animals such as chicken, pig, cattle and sheep [37]. Xu et al. have conducted the first study on microRNAs in chicken in 2006 and they identified 25 microRNAs from chicken embryos and adult chickens [38]. Then many studies have been reported of chicken microRNAs in adipose tissue and skeletal muscle, embryonic chicken livers, chicken embryos, and different stages of gonadal [27, 37, 39]. Hundreds of microRNAs were profiled in chicken embryos and many of them were differentially expressed during embryonic development, suggesting these microRNAs might play vital roles in chicken embryonic development [29]. Furthermore, microRNAs have been considered to act as “buffer” against variation in gene expression [40]. In the face of environmental and genetic perturbations, the biological organisms may use microRNAs to maintain their functions. By reinforcing transcriptional programs and attenuating aberrant transcripts, microRNAs help to confer robustness to biological processes [40]. ZnO NPs have been reported to result in adverse effect on organisms and to change the expression of genes related to cytoskeletal transport, cellular respiration, and reproduction in *Daphnia magna* [16], and in our recent study we found ZnO NPs regulate the protein expression too [41]. Do microRNAs buffer against the variation in gene expression caused by ZnO NPs? It is unknown yet. Although microRNAs might play very important regulatory roles for gene expression and finally protein synthesis in biological systems, they have not been applied in the research related to ZnO NPs yet. Further it will be very meaningful to explore the effects of ZnO NPs on the expression of microRNAs because ZnO NPs have been used in medical applications and microRNAs play vital roles in diseases [2–5, 35, 36]. Therefore the hypothesis of current study was that ZnO NPs might regulate the expression of microRNAs and the correlations of microRNAs and their targeted genes by intact NPs.

As descripted in our previous publication, ovarian granulosa cells of chicken and mammals are similarly steroidogenic hormone production cells and play very important roles in oocyte development and early embryogenesis because they are the closest cells to germ cells and they transport nutrition and produce other factors for oocyte growth [41–44]. Further, our research focus was female reproductive toxicology. Therefore, the chicken ovarian granulosa cells (GCs) were used again as a model in this investigation to explore the effects of ZnO NPs on microRNA expression and the correlation with their target genes in female reproductive systems. ZnSO_4_ was used as a control to provide sole Zn^2+^ effect. It was found that ZnO-NP-5μg/ml specifically regulated the expression of microRNAs involved in development in GCs. And ZnO-NP-5μg/ml also regulated the correlations of microRNAs and their targeted genes. Since ZnO NPs are present everywhere and easily get into human body due to the small size, they might cause adverse effects on our biological systems, even the female reproductive systems.

## Materials and Methods

### ZnO nanoparticles Characterization

The methods for characterization of ZnO NPs were just published in our previous paper [41]. ZnO NPs were synthesized by Beijing DK nano technology Co. LTD (Beijing, P. R. China). The morphology, size, and agglomeration were characterized by transmission electron microscopy (TEM; JEM-2100F, JEOL Inc., Japan) and dynamic light scattering (DLS) particle size analyzer (Nano-Zetasizer-HT, Malvern Instruments, Malvern, UK).

### Granulosa Cells (GC) Isolation and Culture

The protocols were reported in our previous paper [41]. This investigation was carried out in strict accordance with the recommendations in the Guide for the Care and Use of Laboratory Animals of the National Institutes of Health. The protocol was approved by the Committee on the Ethics of Animal Experiments of Qingdao Agricultural University. 30-40weeks old Jinghong-1 laying hens were obtained from Maochangyuan Co. (Qiangdao, China). The hens were terminated, and granulosa cells were isolated from large pre-ovulatory follicles (POFs). Briefly, the sheets containing perivitelline layer and granulosa cell from follicles were carefully separated from yolk. Then the sheets were washed in cold, sterile PBS twice and cut to small pieces. The granulosa cells (GCs) were separated by mechanically pipetting up and down for 2 min. The GCs suspension was filtered and the cell pellets were washed twice by cold PBS and once in blank M199 medium (Cat. No: 31100–019; Gibco^®^, Life Technologies, Carlsbad, CA, U.S.A.) followed by 4-min centrifugation at 250 ×*g* at room temperature. Cell viability of GC was determined to be >95% after isolation. The cells were cultured under standard conditions (37°C, 5% CO2) in M199 medium supplemented with penicillin (50 U/ml), streptomycin (50 mg/ml), 10% fetal bovine serum (FBS; Cat. No: 10099–41; Gibco^®^ Life Technologies), and 1% of Insulin-Transferrin-Selenium-A Supplement (ITS) (Catalog no. 51300–044; Gibco^®^, Life Technologies.).

### Determination of ZnO NPs Effects on Cell Viability

The protocols for determination of cell viability were reported in our previous paper [41]. Briefly, following isolation, GCs were plated in 96-well plates at a density of 5 x 10^4^ cells /well. After two days recovery and growth, GCs were treated with different concentrations (based on Zn) of ZnO NPs or ZnSO_4_ (Cat. No: Z1001, Sigma-Aldrich Co. LLC in China, Beijing, P.R. China) for 24h. After 24h treatments, the cells were washed with fresh basic medium (No FBS or antibiotics) and then cell viability was determined by a colorimetric assay with MTT [3-(4, 5)-dimethylthiazol-2, 5-diphenyltetrazolium bromide; Cat. No: M5655, Sigma-Aldrich] [45].

### Measurement of Zn Concentration in Cultured Cells

The protocols for detection of Zn content in cell were reported in our previous paper [41]. Briefly, isolated GCs were cultured in 6-well plates for 2 days then the cells were treated with different concentration of ZnO NP or ZnSO_4_ for 24h. About 2×10^6^ cells per treatment were collected for determination of Zn in the cells. 500μL of 0.4% Triton X-100 in PBS was used to lyse the cells and the lysate was diluted to 2mL with 0.1% Triton X-100. Samples were analyzed by inductively coupled plasma optical emission spectroscopy (ICP-OES, Optima 2100, Perkin-Elmer, Shelton, CT, USA) [46].

### Detection of ZnO NPs in Cells by Transmission Electron Microscopy (TEM) and Energy Disperse Spectroscopy (EDS)

The protocols for detection of intact NPs in treated cell were reported in our previous paper [41] and other publication [47]. Briefly, ZnO NPs treated GCs pellets were collected and fixed for 2 h in 2% glutaraldehyde made in sodium phosphate buffer (pH 7.2). The specimens were washed extensively to remove the excess fixative and subsequently post-fixed in 1%OsO_4_ for 1h in the dark. After extensive washes in phosphate buffer, the cells were dehydrated in an increasing graded series of ethanol and infiltrated with increased concentration Spur’s embedding medium in propylene epoxide. Then the specimens were polymerized in embedding medium 12h at 37°C, 12h at 45°C and 48h at 60°C. Fifty nanometer were cut on a Leica Ultracut E equipped with a diamond knife (Diatome, Hatfield, PA), and collected on form var-coated, carbon-stabilized molybdenum (Mo) grids. The section-containing grids were stained with uranyl acetate, allowed to air dry overnight, and imaged on a JEM-2010F TEM (JEOL Ltd., Japan). ZnO nanoparticles in the cells were confirmed by X-Max^N^ 80 TLE EDS (Oxford Instruments, U.K.).

### Determination of ZnO-NP Effects on miRNA Expression by Small RNA Sequencing and miRNA q-RT-PCR

#### Small RNA Sequencing

Total RNA was extracted as described in publication [41] Briefly, total RNA was isolated by TRIzol Reagent (Invitrogen, U.S.A.) and purified with PureLink® RNA Mini Kit (Cat: 12183018A; Life Technologies) following the manufacturer’s protocol. And then the samples were gel purified. 18-30nt fragments were selected to build the library. Then two kinds of adapters were ligated to each end of the resulting fragments. The prepared RNA was amplified by reverse transcription PCR (RT-PCR), RT-PCR products were then loaded on Hiseq2000 platform to sequence (Raw sequence reads in S1 Table, S2 Table and S3 Table). The 50nt sequence tags from HiSeq sequencing went through the data cleaning first, which included getting rid of the low quality tags and several kinds of contaminants from the 50nt tags. Length distribution of clean tags was then summarized. Afterwards, the standard bioinformatics analysis was used to annotate the clean tags into different categories and to take those which cannot be annotated to any category to predict the novel miRNA and base edit of potential known miRNA [48].

#### miRNA q-RT-PCR

miRNA q-RT-PCR was performed by the Hairpin-it^TM^ miRNA RT-PCR (probe) Quantitation kit from GenePharma Co., Ltd (Shanghai, P.R. China) following the manufacturer’s instruction. One microgram of total RNA was used to make the first strand cDNA: 4 μl 5x RT buffer, 0.75μl 10mM dNTP, 1.20μl 1μM miRNA and 5s rRNA RT primer mix, 0.2 μL reverse transcriptase (200U/μl), 1μg RNA sample and RNase free water to 20μl. The program for the reaction was 25°C 30min, 42°C 30min, 85°C 5min, then 4°C or on ice. The generated first-strand cDNAs, 2μl/sample, was diluted to 20μl with ddH2O for 5s rRNA q-PCR. Then, 2μl of the RT product was used for one PCR reaction (in a 96-well plate) for each miRNA (Three replications for every sample). Each PCR reaction (20μl) contained 10μl of 2x qPCR Master Mix (FAM), 0.4μl miRNA/5s rRNA specific primer set (10μM), 0.2μl of miRNA/5s rRNA specific probe (10μM), 0.2μl Taq DNA polymerase (5U/μl), 2μl RT product and ddH2O to 20μL. The qPCR was performed with the Roche LightCycler^®^ 480 (Roche, German) and the reaction was as following, step 1: 95°C, 3 min; step 2: 40 cycles of 95°C, 12 s; 62°C, 40sec. Three or more independent experiment samples were analyzed.

## MicroRNAs Targeted Genes Prediction

The data for RNA-seq transcript profiling of ZnO-NP-5μg/ml and ZnSO4-10μg/ml treated GCs were just published in our recent paper [41]. MicroRNA targeted mRNA have been predicted using mirbase, miranda and mirdb and plugged within GeneSpring GX software. The final results were predicted from the union part of the three databases. The data of significantly expressed microRNAs and mRNAs were analyzed in this study. The correlations of microRNA and mRNAs were present as up-up, up-down, down-up and down-down (microRNAs in front).

## Statistical Analyses

The q-RT-PCR was statistically analyzed using proprietary software from SABiosciences online support. Other results were expressed as mean ± SEM. Differences were considered significant at *p* < 0.05 using ANOVA.

## Results

### Characteristics of ZnO NPs

**Fig 1:**
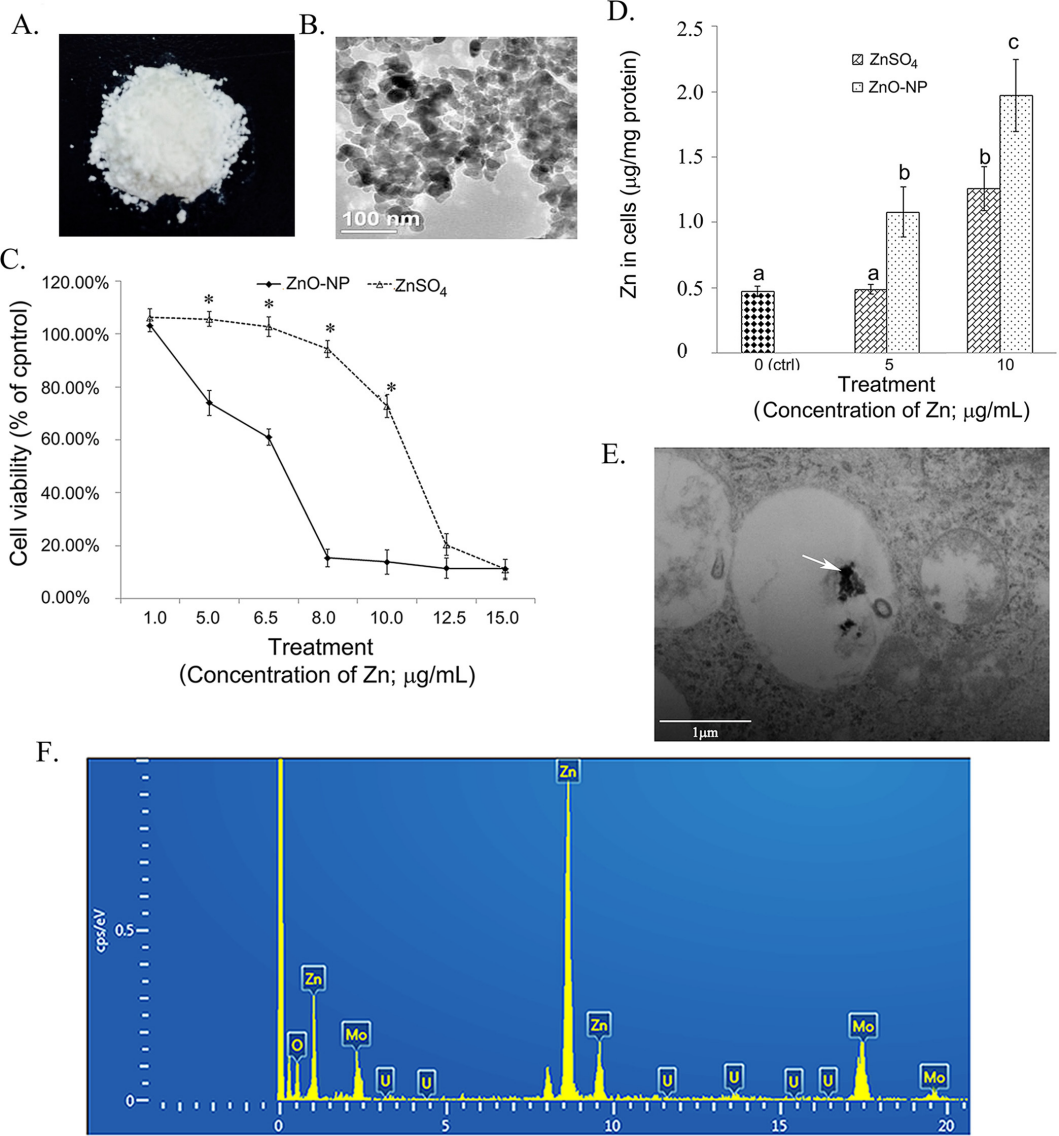
Effects of ZnO NPs on cell growth and intracellular concentration of Zn. A. Photo of ZnO NPs; B. TEM image of ZnO NPs. C. Effects of ZnO NP and ZnSO4 on GCs growth, * p<0.05; D. Concentration of Zn in ZnO NPs and ZnSO4 treated GCs, a,b,c, same letter meant no difference, different letters indicated the difference between groups, p<0.05; E. TEM image of ZnO NPs in GCs indicated by the white arrow. F. EDS picture of ZnO NPs in GCs, where three Zn peaks shown.

The photo of ZnO NPs used in this investigation was in Fig 1A, and the ultra-structures of NPs (analyzed by TEM) were shown in Fig 1B. As reported in our previous publication [41], the color, density, size and surface area of spherical ZnO NPs used in our studies were milk white, 30nm, 50m^2^/g and 5.606g/cm^3^.

### Effect of ZnO NPs on the growth of granulosa cells (GCs)

The growth, the basic effect of ZnO NPs on granulosa cells, was investigated firstly. As shown in Fig 1C, both ZnO NPs and ZnSO_4_ inhibited the growth of GCs after 24h treatment. However, ZnO NPs caused higher cell growth inhibition than ZnSO_4_. The IC_50_ for ZnO NPs was 7.3μg/ml however IC50 for ZnSO_4_ treatment was11.1μg/ml [41]. The concentration was calculated based on the concentration of Zn. Similar growth inhibition was produced by ZnO-NP-5μg/ml and ZnSO_4_-10μg/ml treatments.

### Concentration of Zn in GCs

As shown in Fig 1D, the concentrations of Zn in GCs after 24h treatment were different when the same concentration of ZnO NPs and ZnSO_4_ were used. The concentration of Zn in ZnSO_4_-5μg/ml treated GCs was similar to that in the control cells. The concentrations of Zn in ZnO-NP-5μg/ml and ZnSO_4_-10μg/ml treated GCs were almost the same suggesting that NPs were absorbed into cells more readily than Zn^2+^. The concentration of Zn in ZnO-NP-10μg/ml treated GCs was much higher than that in ZnSO_4_-10μg/ml treated cells. The two treatments ZnO-NP-5μg/ml and ZnSO_4_-10μg/ml were used in this study to explore the effects of ZnO NPs or Zn^2+^ on miRNA expression in GCs because these two treatments produced same intracellular Zn concentration and resulted in similar cell growth inhibition [41].

### ZnO NPs in GCs

Next question was that whether there were any intact NPs in ZnO NPs treated cells? After 24h ZnO-NP-5μg/ml treatment, TEM was used to analyze the NPs in the treated cells. NPs were present in the treated cells (Fig 1E; indicated by white arrow). The NPs were in membrane-bound vesicles. The NPs in the cells were confirmed by Energy Disperse Spectroscopy (EDS) with Zn (Fig 1F) where three standard Zn peaks were present [41].

### Effects of ZnO NPs on the expression of microRNAs

**Fig 2:**
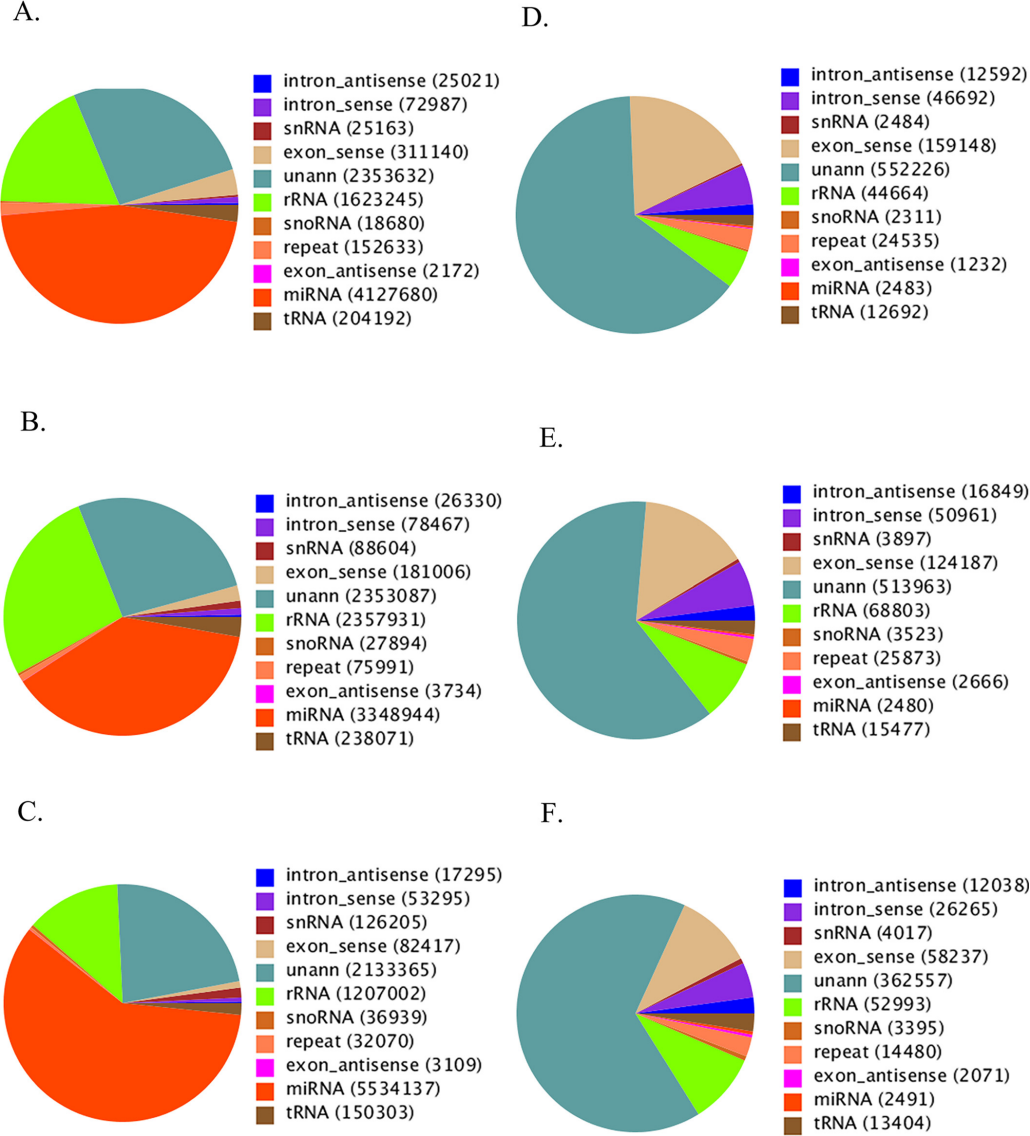
Distribution of small RNA among different categories. Eleven categories: exon-antisense, exon-sense, intron-antisense, intron-sense, microRNA, rRNA, repeat, snRNA, snoRNA, tRNA and unann. A. Pie chart for annotation of small RNA in control GCs (total for each category); B. Pie chart for annotation of small RNA in ZnO-NP-5μg/ml treated GCs (total for each category); C. Pie chart for annotation of small RNA in ZnSO_4_-10μg/ml treated GCs (total for each category); D. Pie chart for annotation of small RNA in control GCs (unique for each category); E. Pie chart for annotation of small RNA in ZnO-NP-5μg/ml treated GCs (unique for each category); F. Pie chart for annotation of small RNA in ZnSO_4_-10μg/ml treated GCs (unique for each category).

Could NPs have any specific effects on the expression of microRNAs in ZnO NPs treated GCs since NPs were found in these cells? ZnO NPs specifically regulated the expression of microRNAs. Fig 2A, 2B and 2C presented the distribution of small RNAs (total number) among different categories, and Fig 2D, 2E and 2F showed the distribution of unique expressed small RNAs among different categories in control, ZnO-NP-5μg/ml and ZnSO_4_-10μg/ml treated GCs, respectively. The numbers of annotated total microRNAs were most in ZnSO_4_-10μg/ml treated GCs, then in control GCs, and the least in ZnO-NP-5μg/ml treated GCs (Fig 2A, 2B and 2C). However, there was no big difference for the number of unique expressed microRNA for these three treatments (Fig 2D, 2E and 2F).

**Fig 3:**
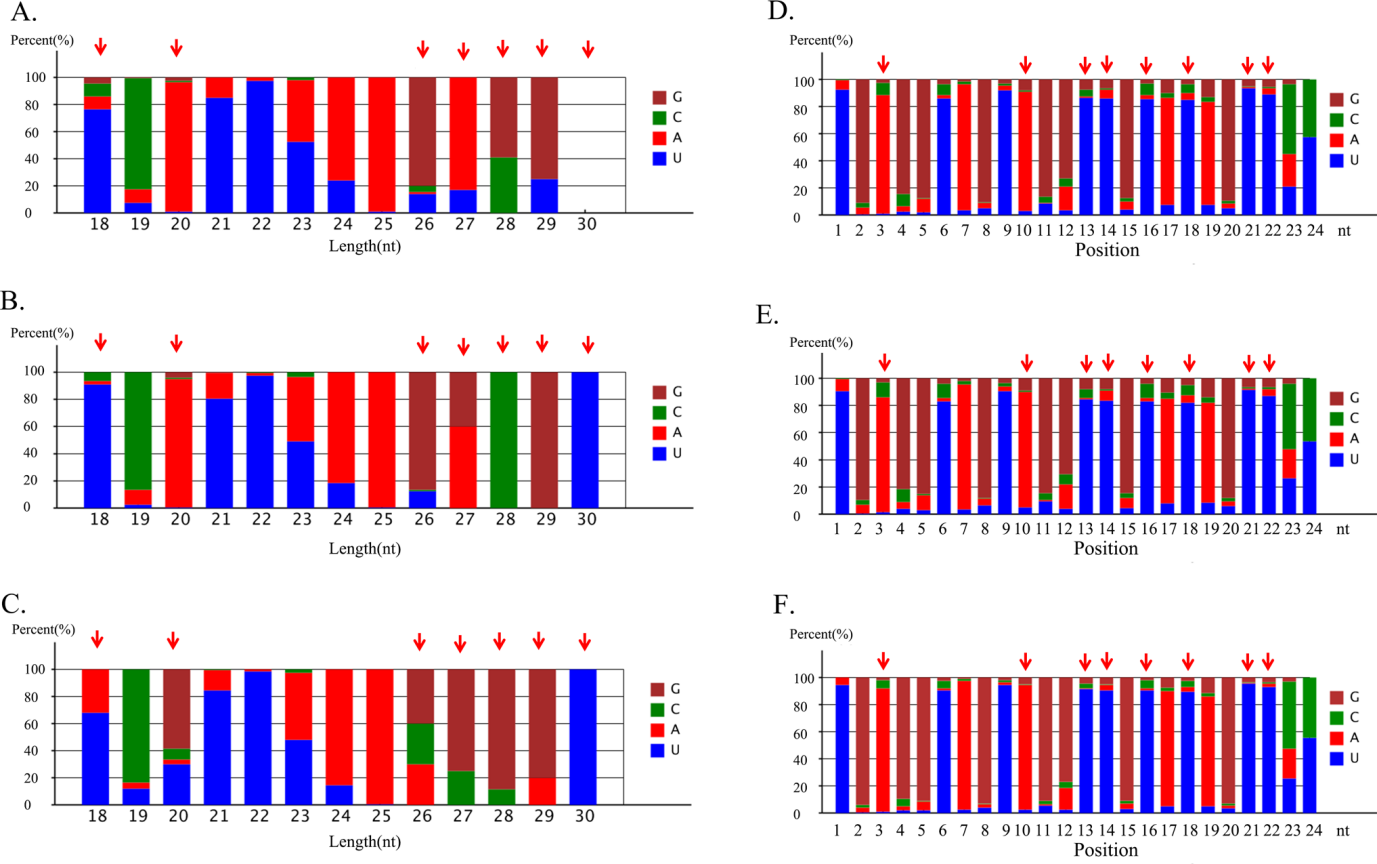
Summary of known microRNA nucleotides bias. A. microRNA first nucleotide bias in control GCs; B. microRNA first nucleotide bias in ZnO-NP-5μg/ml treated GCs; C. microRNA first nucleotide bias in ZnSO_4_-10μg/ml treated GCs; D. microRNA nucleotide bias at each position in control GCs; E. microRNA nucleotide bias at each position in ZnO-NP-5μg/ml treated GCs; F. microRNA nucleotide bias at each position in ZnSO_4_-10μg/ml treated GCs.

The length distribution of known microRNAs in this study was from 18 to 30nt, and most of them were about 20-24nt. The first nucleotide of known microRNAs (18-30nt length) was different among control, ZnO-NP-5μg/ml and ZnSO_4_-10μg/ml treatment (Fig 3A, 3B and 3C). The big differences between these three treatments were for 18nt, 20nt, 26nt, 27nt, 28nt, 29nt and 30nt length microRNAs (indicated by red arrow). The nucleotides at each position in the microRNAs were also different between the three treatments (Fig 3D, 3E, and 3F). And the big differences between these three treatments were for nucleotides at the position 3, 10, 13, 14, 16, 18, 21 and 22 (indicated by red arrow).

**Fig 4:**
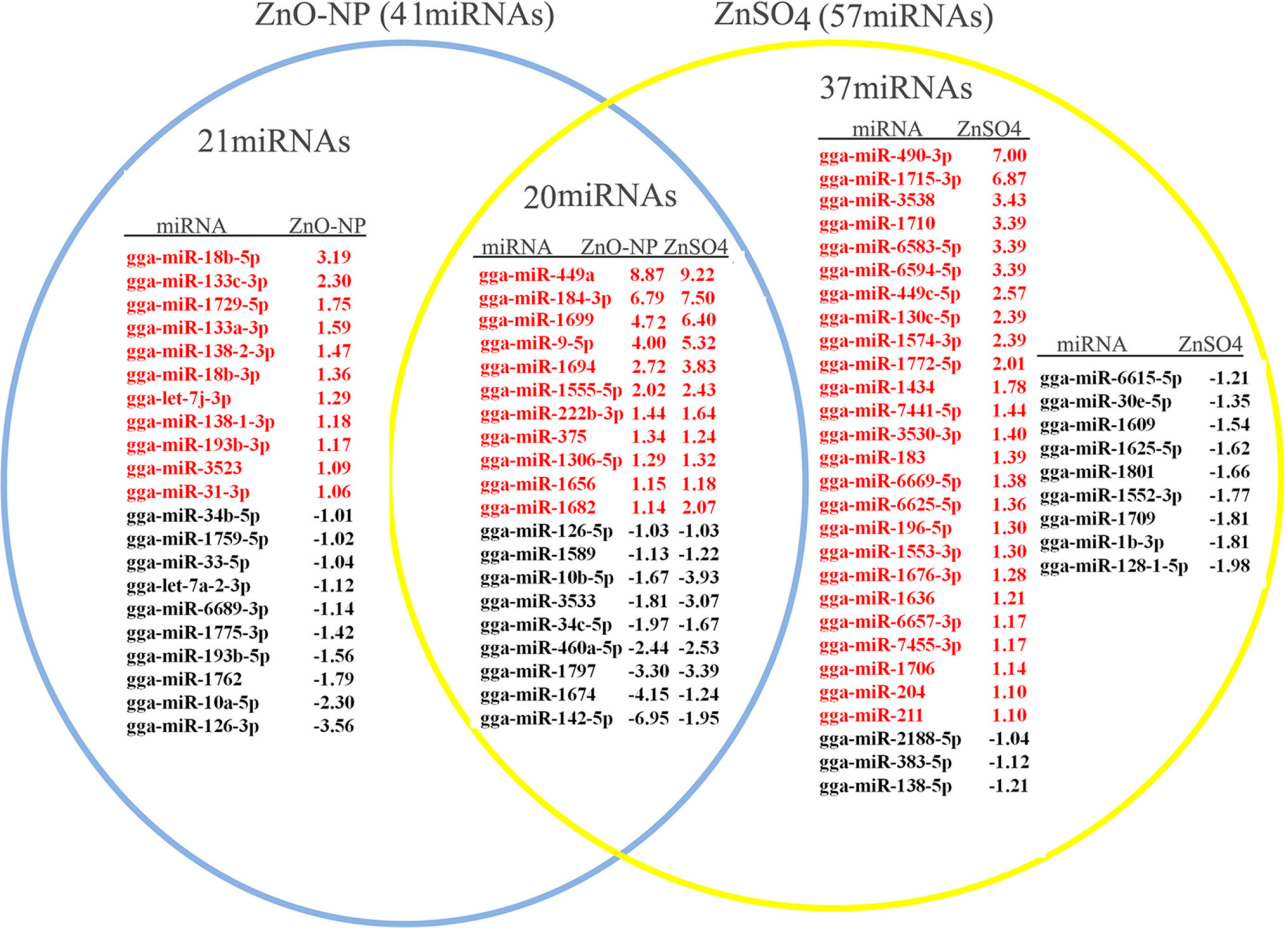
List of total microRNAs significantly regulated by ZnO-NP-5μg/mL and/or ZnSO4-10μg/mL: 20 microRNAs regulated by the two treatments, 21 microRNAs regulated by ZnO-NP-5μg/ml, and 37 microRNAs regulated by ZnSO_4_-10μg/ml. The number was the log_2_Ratio value, cut off at log_2_Ratio ≥1 orlog_2_Ratio ≤-1. They were significantly regulated.

**Fig 5:**
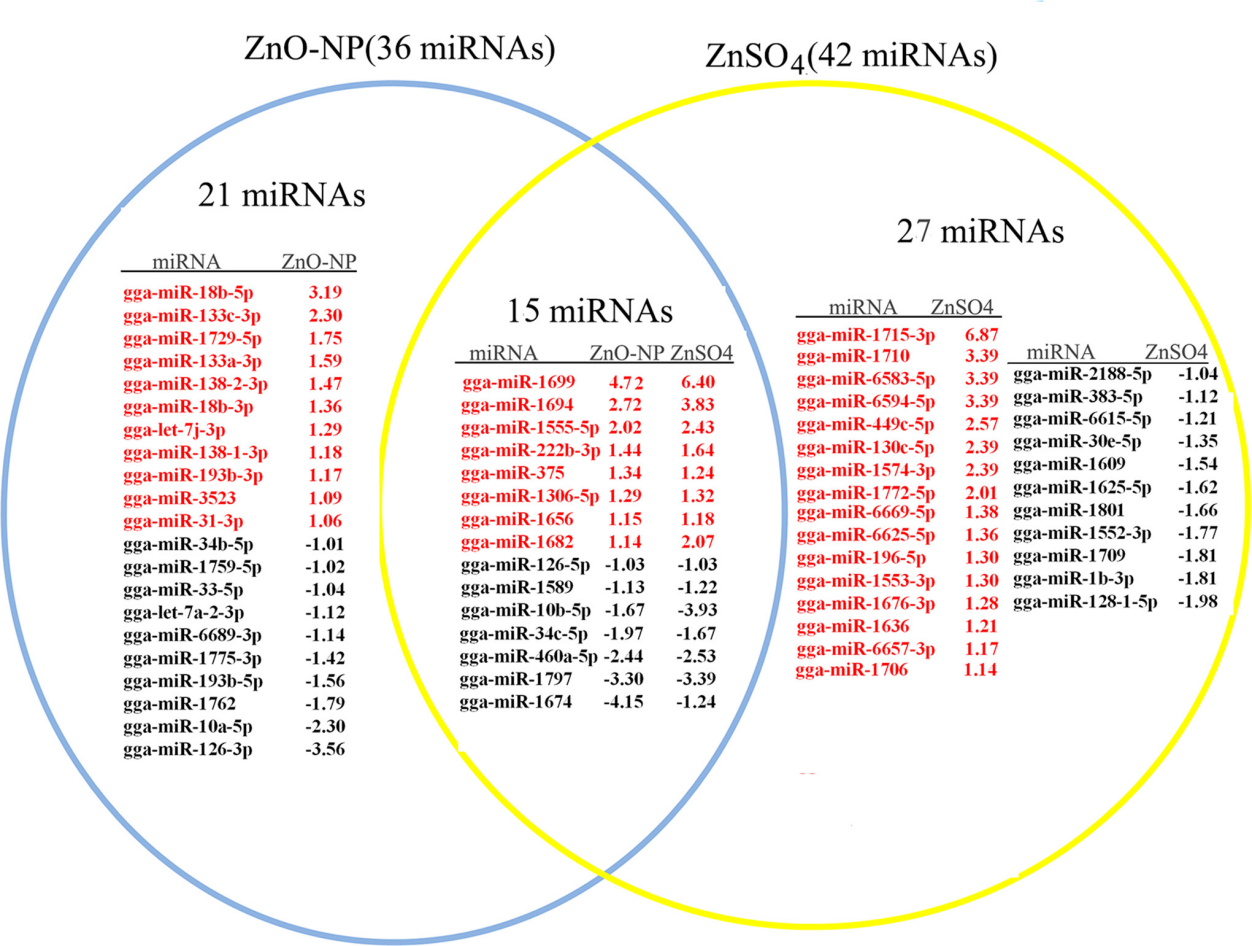
The significantly regulated microRNAs involved in embryonic development: 15 of them regulated by the two treatment groups, 21 of them regulated just by ZnO-NP-5μg/mL and 27 of them regulated only by ZnSO4-10μg/mL. The number was the log_2_Ratio value, cut off at log_2_Ratio ≥1 orlog_2_Ratio ≤-1. They were significantly regulated.

**Fig 6:**
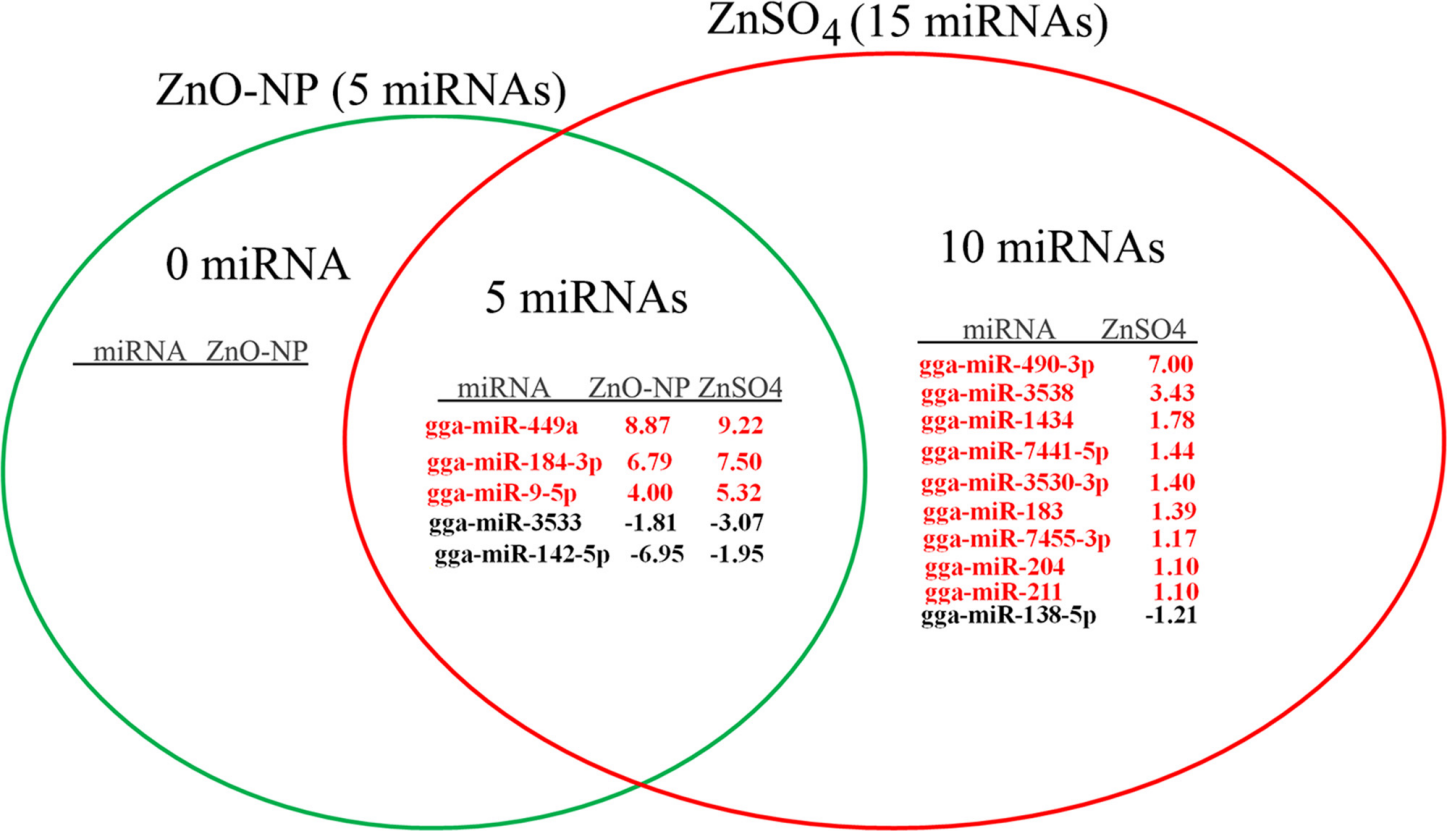
The regulated miRNAs related to revolution: 5 of them regulated by the two treatment groups, and 10 of them regulated only by ZnSO4-10μg/mL. The red color meaning the microRNAs up regulated, black meaning microRNAs down regulated. The number was the log_2_Ratio value, cut off at log_2_Ratio ≥1 orlog_2_Ratio ≤-1. They were significantly regulated.

Seventy eight known microRNAs were differentially altered by ZnO NPs and/or ZnSO_4_ (half of the microRNAs up-regulated) (Fig 4, red color indicated up regulation, black color meant down regulation; S4 Table). The expression model cluster analysis for the known microRNAs was present in S1 Fig Of the 78 known microRNAs regulated by ZnO-NP-5μg/ml and/or ZnSO_4_-10μg/ml, 41 microRNAs were altered in ZnO-NP-5μg/ml treatment; 57 microRNAs were changed by ZnSO_4_-10μg/ml (Fig 4). Of the regulated 78 microRNAs, 63 microRNAs are involved in development (most in embryonic development) (Fig 5); 15 microRNAs are related to revolution (Fig 6). Of the 21 microRNAs specifically regulated by ZnO-NP-5μg/ml, all of them are related to development (Fig 5). However of the 37 microRNAs specifically altered by ZnSO_4_-10μg/ml, only 73.0% (27/37) are involved in development (Fig 5). The 20 microRNAs altered by both ZnO-NP and ZnSO_4_, 15 are involved in development (Fig 5). Since ZnO-NP-5μg/ml regulated own specific sets of microRNAs and NPs were found in ZnO NPs treated GCs, these together documented that NPs had specific regulatory roles on the expression of microRNAs compared to Zn^2+^.

MicroRNA q-RT-PCR analysis was used to confirm the small RNA sequencing results. Four microRNAs (miRNA-18b-5p, 193b-3p, 133a-3p and 133c-3p) related to embryonic development were analyzed by q-RT-PCR (Table 1). The q-RT-PCR data matched well with small RNA sequencing data.

**Table 1:** MicroRNA q-RT-PCR data.

microRNA	ZnO-NP-5		ZnSO_4_-10	
Name	Fold Change	p-value	Fold Change	p-value
gga-miR-18b-5p	**1.74**	0.045	**—**	
gga-miR-193b-3p	**1.73**	0.032	**—**	
gga-miR-133a-3p	**3.63**	0.012	**—**	
gga-miR-133c-3p	**3.42**	0.022	**—**	

### Effects of ZnO NPs on the correlations of microRNAs and their targeted genes

**Fig 7:**
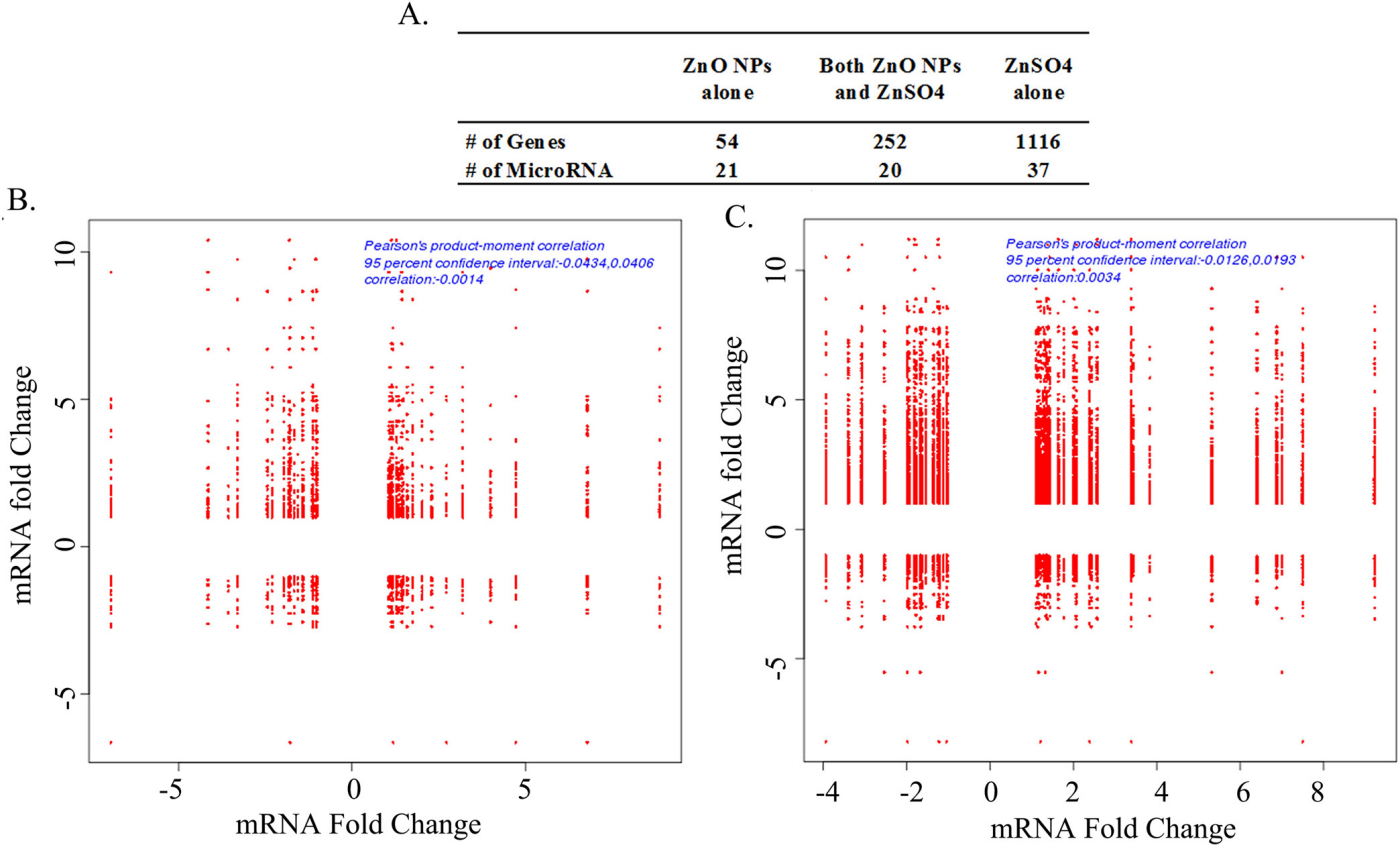
Correlations of microRNAs and their targeted genes. A. Summary of the significantly regulated microRNAs and mRNAs; B. The correlations of microRNAs and mRNAs regulated by ZnO-NP-5μg/mL treatment compared to control, x-axis was the fold change of microRNA, y-axis was the fold change of mRNA; B. The correlations of microRNAs and mRNAs regulated by ZnSO4-10μg/mL treatment compared to control, x-axis was the fold change of microRNA, y-axis was the fold change of mRNA.

**Fig 8:**
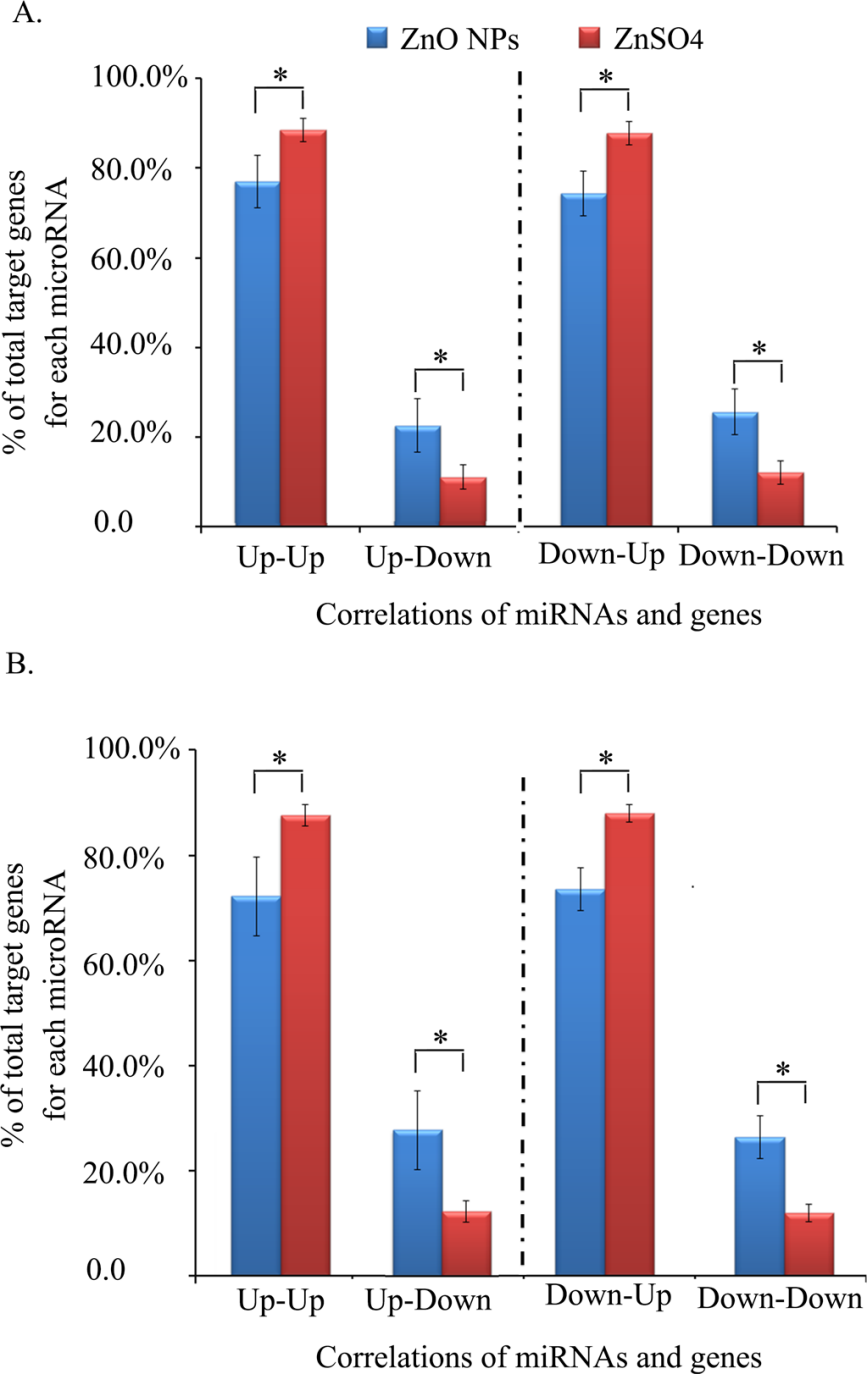
Percentage of the up or down regulated targeted genes compared to the total targeted genes of each microRNA. A. Percentage of the up or down regulated targeted genes compared to the total targeted genes of each microRNA altered by ZnO-NP-5μg/ml treatment alone or ZnSO_4_-10μg/ml treatment alone; B. Percentage of the up or down regulated targeted genes compared to the total targeted genes of each microRNA altered by both ZnO-NP-5μg/ml and ZnSO_4_-10μg/ml treatments. Up-UP meant that both microRNAs and mRNAs were up regulated. Up-Down indicated that microRNAs were up regulated and mRNAs were down regulated. Down-Up indicated that microRNAs were down regulated and mRNAs were up regulated. Down-Down indicated that both microRNAs mRNAs were down regulated.

The RNA-seq transcript profiling data of ZnO-NP-5μg/ml and ZnSO4-10μg/ml treated GCs were just published in our paper [41]. Fig 7A summarized the numbers of differently expressed microRNAs and mRNAs by ZnO-NP-5μg/ml alone, ZnSO_4_-10μg/ml alone and both of these two treatments. The correlations of microRNAs and their targeted genes were analyzed (S5 Table and S6 Table). Only the significant expressed known microRNAs or mRNAs by ZnO-NP-5μg/ml and/or ZnSO_4_-10μg/ml treatments were analyzed. The correlations were present as up-up, up-down, down-up and down-down (microRNAs in front). Fig 7B and 7C showed the correlations of the significantly expressed microRNAs and their targeted genes (Fig 7B for ZnO-NP-5μg/ml treatment; Fig 7C for ZnSO_4_-10μg/ml treatment). Table 2 listed the numbers of targeted genes for significant expressed microRNAs. The numbers in the italic and bold formatting meant the microRNAs were up-regulated and others were down-regulated. More targeted genes were found in ZnSO_4_-10μg/ml treatment than that in ZnO-NP-5μg/ml treatment. The data for the comparation of the up or down regulated targeted genes to the total targeted genes of each microRNA were shown in Fig 8. And Fig 8A presented the data for ZnO-NP-5μg/ml alone or ZnSO_4_-10μg/ml alone altered microRNAs. The percentage of up-regulated targeted genes (up-up or down-up) was lower in ZnO-NP-5μg/ml treatment than that in ZnSO_4_-10μg/ml treatment however the percentage of down-regulated targeted genes (up-down or down-down) was higher in ZnO-NP-5μg/ml treatment than that in ZnSO_4_-10μg/ml treatment (Fig 8A). The similar trend was for the microRNAs regulated by both ZnO-NP-5vg/ml and ZnSO_4_-10μg/ml treatments (Fig 8B). The data suggested that ZnO NPs also specifically regulated the correlations of microRNAs and their targeted genes.

**Table 2:** The number of target genes for each microRNA.

ZnO NPs			Both		ZnSO4	
			ZnO NPs	ZnSO4		
MicroRNA	# of target genes	MicroRNA	# of target genes	# of target genes	MicroRNA	# of target genes
*gga-miR-18b-5p*	***62***	*gga-miR-449a*	***65***	***295***	*gga-miR-490-3p*	***223***
*gga-miR-133c-3p*	***48***	*gga-miR-184-3p*	***59***	***252***	*gga-miR-1715-3p*	***220***
*gga-miR-1729-5p*	***57***	*gga-miR-9-5p*	***35***	***173***	*gga-miR-3538*	***183***
*gga-miR-133a-3p*	***47***	*gga-miR-1694*	***24***	***86***	*gga-miR-1710*	***320***
*gga-miR-138-2-3p*	***48***	*gga-miR-1555-5p*	***34***	***207***	*gga-miR-6583-5p*	***244***
*gga-miR-18b-3p*	***37***	*gga-miR-222b-3p*	***68***	***307***	*gga-miR-6594-5p*	***299***
*gga-miR-7j-3p*	***55***	*gga-miR-375*	***8***	***33***	*gga-miR-449c-5p*	***248***
*gga-miR-138-1-3p*	***67***	*gga-miR-1306-5p*	***57***	***241***	*gga-miR-130c-5p*	***260***
*gga-miR-193b-3p*	***47***	*gga-miR-1656*	***44***	***191***	*gga-miR-1574-3p*	***282***
*gga-miR-3523*	***37***	*gga-miR-1682*	***74***	***289***	*gga-miR-1772-5p*	***389***
*gga-miR-31-3p*	***55***	*gga-miR-1699*	***62***	***265***	*gga-miR-1434*	***214***
gga-miR-34b-5p	92	gga-miR-126-5p	44	199	*gga-miR-7441-5p*	***349***
gga-miR-1759-5p	34	gga-miR-1589	83	431	*gga-miR-3530-3p*	***235***
gga-miR-33-5p	35	gga-miR-10b-5p	41	210	*gga-miR-183*	***285***
gga-miR-7a-2-3p	85	gga-miR-3533	88	421	*gga-miR-6669-5p*	***67***
gga-miR-6689-3p	43	gga-miR-34c-5p	72	338	*gga-miR-6625-5p*	***407***
gga-miR-1775	97	gga-miR-460a-5p	49	277	*gga-miR-196-5p*	***181***
gga-miR-193b-5p	22	gga-miR-1797	57	271	*gga-miR-1553-3p*	***164***
gga-miR-1762	67	gga-miR-1674	45	196	*gga-miR-1676-3p*	***163***
gga-miR-10a-5p	43	gga-miR-142-5p	73	353	*gga-miR-1636*	***224***
gga-miR-126-3p	19				*gga-miR-6657-3p*	***182***
					*gga-miR-7455-3p*	***196***
					*gga-miR-1706*	***164***
					*gga-miR-204*	***249***
					*gga-miR-211*	***249***
					gga-miR-2188-5p	511
					gga-miR-383	331
					gga-miR-138-5p	417
					gga-miR-6615-5p	272
					gga-miR-30e-5p	377
					gga-miR-1609	338
					gga-miR-1625-5p	147
					gga-miR-1801	328
					gga-miR-1552-3p	338
					gga-miR-1709	266
					gga-miR-16-3p	332
					gga-miR-128-1-5p	411

## Discussion

The goals of this study were to explore the effects of ZnO NPs on the expression of microRNAs and the correlation of microRNA and their targeted genes to unveil the underline molecular insights of ZnO NPs on biological systems especially on female reproductive systems. Our results demonstrated that ZnO NPs were different from ZnSO_4_. More than 5μg/ml of ZnSO_4_ gradually inhibited the growth of GCs. However, ZnO NPs sharply inhibited the growth of GCs started at 1μg/ml. The concentration of Zn in ZnO-NP-5μg/ml and ZnSO_4_-10μg/ml treated GCs was almost the same, and these two treatment inhibited similar cell growth. This was supported by a previous report that NPs were easily absorbed into biological systems because the small size [49]. Also, as reported in our previous publication [41], NPs were identified in the treated cells and confirmed by EDS. The mechanisms of the internalization of ZnO NPs into biological systems are not fully understood. Endocytosis is considered as the major route for the uptake of NPs int biological system [24, 50, 51]. And it has also been reported that the uptake of ZnO NPs can be mediated by M cells (specialized phagocytic enterocytes) and normal intestinal enterocytes in the intestinal tract [24, 50]. Furthermore, the protein-nanoparticles interactions facilitate the delivery of nanoparticles to organs, because plasma proteins play vital roles in the disposition and transportation of both endogenous and exogenous molecules [50, 52, 53].

Although the two treatments resulted in same intracellular Zn concentrations and similar cell growth inhibition, ZnO-NPs-5μg/ml differentially affected the expression of microRNAs and the correlations of microRNAs and their targeted genes compared to ZnSO_4_-10μg/ml even though these two treatment inhibited similar cell growth, and the content of Zn was also almost the same in these treatments. Most of the changed microRNAs are involved in development (especially in the embryonic development) and some examples of them are miRNA-222, miRNA-383, miRNA-126, miRNA-133, miRNA-30, miRNA-10a, miRNA-196 and miRNA-18b. The detailed functions of the microRNAs in the development have not been defined clear yet. However, it has been found that the target pathways of microRNAs in early embryo included Wnt, TGF-β (transforming growth factorβ), MAPK (mitogen-activated protein kinase) and mTOR (mammalian target of rapamycin) signaling pathways [26]. MicroRNAs might govern the genes in these pathways to regulate the embryo development. And the functions of a few of microRNAs have been investigated extensively. For examples, miRNA-222 is known as the regulator of kit ligand signaling during the recruitment and maintenance of precursor hematopoietic cells and it is involved in the regulation of cholesterol synthesis in embryonic liver [27]; and miR-133 has a number of effects on muscle cells including preventing skeletal differentiation, enhancing myoblast proliferation by repressing serum response factor (SRF) [54]. Fifteen known microRNA altered by ZnO-NP-5μg/ml and/or ZnSO_4_-10μg/ml are related to evolution including miRNA-7441-5p, miRNA-7455, miRNA-183, miRNA-211, and miRNA-204 and others [38, 55].

The “buffer” effects of microRNAs have been considered as a mode of action of them. That microRNAs were found to be globally depleted in tumors relative to their normal tissue counterparts was the example of microRNAs as general mutation buffering agents [40]. These depletions included the knockdown of components of the microRNA biogenesis pathway [56–58] or heterozygous deletion of Dicer [48]. ZnO-NP-5μg/ml specifically altered the expression of certain microRNAs involved in embryonic development and ovarian functions and it also specifically regulated the correlations of microRNAs and their targeted genes. This suggested that NPs in the treated cell might perturb the expression of genes, and as the regulatory systems, microRNAs tried to correct the perturbations by reinforcing transcriptional programs and attenuating aberrant transcripts.

In conclusion, microRNAs play vital roles in our biological systems and this is the first investigation of microRNAs involved in ZnO NPs’ toxicological effects on GCs. Intact NPs in ZnO-NP-5μg/ml treatment specifically regulated the expression of microRNAs, and the correlations of microRNAs and their targeted genes compared to that by Zn^2+^. The changed miRNA or genes by NPs might pose adverse effects on female reproductive systems through altered some specific signaling pathways. This expands our knowledge for biological effects of ZnO NPs. Further mechanistic studies on how ZnO NPs specifically regulated microRNAs are warranted.

## Supporting Information

S1 Fig The expression model cluster analysis for the known microRNAs regulated by ZnO-NP-5 μg/ml and/or ZnSO_4_-10μg/ml treatments. (TIF)

S1 Table Raw sequence reads of small RNA sequencing analysis for control treatment (length, reads, sequence). (TXT)

S2 Table Raw sequence reads of small RNA sequencing analysis for ZnSO_4_-10μg/kg treatment (length, reads, sequence). (TXT)

S3 Table Raw sequence reads of small RNA sequencing analysis for ZnO-NP-5μg/kg treatment (length, reads, sequence). (TXT)

S4 Table Raw data of small RNA sequencing analysis. (XLS)

S5 Table The microRNAs and targeted genes correlations analysis for ZnO-NP-5μg/ml treatment. (XLS)

S6 Table The microRNAs and targeted genes correlations analysis for ZnSO_4_-10μg/ml treatment. (XLS)
