# Supplementary figures and images for: Regulation of MicroRNAs, and the Correlations of MicroRNAs and Their Targeted Genes by Zinc Oxide Nanoparticles in Ovarian Granulosa Cells
Source: PLoS One. 2016 May 19;11(5):e0155865. doi: 10.1371/journal.pone.0155865 (PMC4873213; doi:10.1371/journal.pone.0155865)

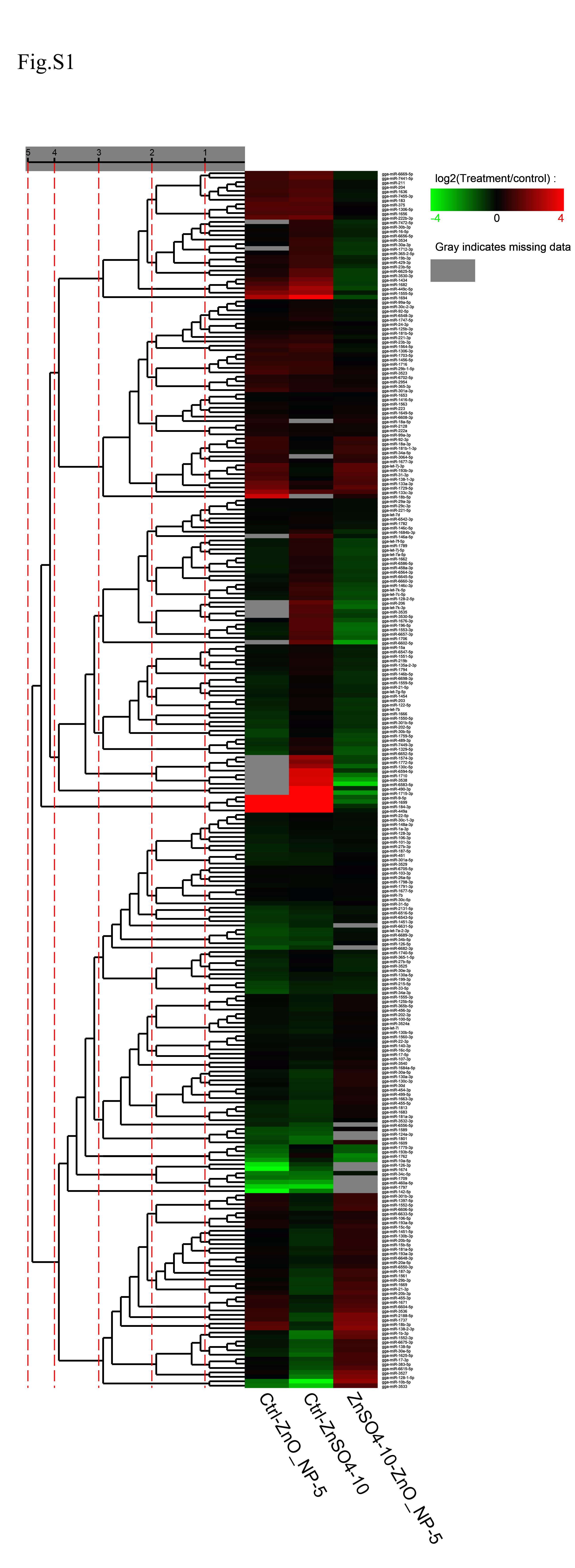

Supplement: S1 Fig — (TIF) [file pone.0155865.s001.tif]
